# Endodontic Access Cavity Design and Fracture Resistance: A Systematic Review and Meta-Analysis of Conventional vs. Newer Access Cavity

**DOI:** 10.7759/cureus.68796

**Published:** 2024-09-06

**Authors:** Mrinalini Mrinalini, Alpa Gupta, Sonal Soi, Dax Abraham, Seema H Bukhari

**Affiliations:** 1 Conservative Dentistry and Endodontics, Manav Rachna Dental College, Faridabad, IND

**Keywords:** conservative endodontic access, conventional endodontic access, fracture resistance, traditional endodontic access, truss endodontic access

## Abstract

The era of minimally invasive dentistry has led to the development of new access cavity designs. The impact of various access cavity designs on the fracture resistance of teeth has been extensively studied. The primary aim of this systematic review and meta-analysis is to evaluate and compare the effects of recent modifications in endodontic access cavity design- specifically, conventional, conservative, and truss designs on tooth fracture resistance. Three independent reviewers searched studies across six different databases (PubMed, Scopus, EBSCOhost, BVS, Wiley, and Google Scholar) from January 2000 to July 2024, following Preferred Reporting Items for Systematic Reviews and Meta-Analyses (PRISMA) guidelines. The articles were then screened using strict inclusion and exclusion criteria. A quality assessment was performed using a modified version of the quality assessment of in-vitro studies according to the QUIN (Quality Assessment Tool For In Vitro Studies) tool, categorizing the selected articles into low, moderate, and high risk of bias. Quantitative data synthesis was conducted to combine equivalent results using STATA. Forest plots were created with the level of significance set at 0.05 (p = 0.05). Out of 243 articles, 14 met the strict inclusion criteria. Among the selected articles, 11 showed a low risk of bias and three showed a moderate risk. The meta-analysis revealed that fracture resistance of conservative and truss access designs is significantly higher than that of conventional endodontic access, with a standardized mean difference (SMD) of 2.61 (95% 1.47 to 3.74; p-values <0.001) and SMD = -1.26 (95% confidence interval (CI): -1.81 to 0-0.71; p<0.001). The heterogeneity (I²) values for these comparisons were 92% and 65.6%, respectively. The extent of the access cavity has a substantial impact on tooth fracture resistance. Newer conservative and truss endodontic access designs offer better fracture resistance compared to conventional endodontic access.

## Introduction and background

Access cavity preparation is the key to successful orthograde endodontic treatment [1]. The “ideal” access cavity typically described in endodontic textbooks usually depicts easily identifiable canal entrances at the base of a large pulp floor. For many years, access cavity was practiced by removing the entire roof of the pulp chamber to better visualize the pulpal floor, as well as to ensure canal identification, straight-line access, shaping, irrigation, and obturation [2]. Meanwhile, numerous studies have demonstrated that this kind of access cavity results in a significant loss of tooth structure, lowering the fracture resistance of the treated teeth [3].

Earlier endodontic access cavities used to be standardized based on the type of tooth, but with technological advancement in endodontic techniques, 3D diagnostic procedures, incorporation of the dental operating microscope, and loupes that provide magnification and better illumination, the cavity design is now primarily determined by the unique pulp chamber morphology of the tooth being treated [4]. In physiologically young teeth, the pericervical dentin (PCD), undermined dentin, dentinoenamel junction (DEJ), axial wall of the DEJ, and cervical enamel, which have been deemed of high value with regard to tissue type, becomes crucial to maintain appropriate strength and fracture resistance [5].

For the past few decades, the need for the conservation of tooth structure has been increasing in dentistry, especially in the preventive and restorative parts of dental treatment. Minimally invasive dentistry has given rise to contracted endodontic cavities (CECs). They’ve been put out as an alternative to conventional endodontic access cavities intended to maintain the mechanical integrity of the tooth [6, 7].

Conservative endodontic cavity preparation techniques designed to minimize the removal of tooth structure and preserve the pulp chamber roofs and PCD have been documented in the literature [8]. However, the restricted cleaning, shaping, and filling of the root canals due to the constrained access cavity outline increases the possibility of ineffective canal instrumentation and the likelihood of procedural mistakes. It is necessary to conduct specific research on conservative endodontic cavity preparation to determine the risks and advantages for various tooth types [9, 10, 11].

The fundamental implication of the truss approach is the preservation of the residual tooth structure, which involves direct access from the occlusal surface to expose the mesial and distal canal orifices and keep the intervening dentin intact. It is said to lessen the need for complicated, more expensive post-endodontic restorations and increase the fracture resistance of teeth that have had endodontic treatment [12].

The effect of various endodontic access cavity designs on fracture resistance and other properties has been thoroughly researched. To provide a careful synthesis of the results, a systematic review of related studies and a meta-analysis are required. The present systematic review compares fracture resistance of conservative and truss access cavities with traditional endodontic access. Since the data obtained through the systematic review was in the form of Mean ± SD, meta-analysis was also applied.

## Review

Materials and methods

Protocol Registration

Following the Preferred Reporting Items for Systematic Reviews and Meta-Analyses (PRISMA) criteria, a systematic review of literature on minimal endodontic access cavities was carried out. It was further registered in the Open Science Framework (OSF) (https://osf.io/zmh6p)

Research Question

The PICOS (Population, Intervention, Comparison, Outcomes, and Study) framework was used to develop the research question, which defined the population, intervention, control group, and outcome.

Is the fracture resistance influenced by variation in the design of the endodontic access cavity in molar teeth when assessed in in-vitro studies? (P= extracted mandibular molar teeth, I= conservative and truss access, C=conventional access, O= fracture resistance, S=in-vitro and ex-vivo studies)

Literature Search Study

The exploration of relevant literature involved searches across six databases - PubMed, Scopus, EBSCOHost, BVS, Wiley, and Google Scholar described from January 2000 to July 2024 restricted to the English language only. Mesh terms were employed and the search strategy was constructed using Boolean operators (“AND” and “OR”). The search data is described in Table 1. The three examiners scrutinized the search database, and inclusion or exclusion decisions were based on specific criteria.

To find the pertinent studies for inclusion in this review, two independent reviewers (M.M. and A.G.) searched through six electronic databases from January 2000 to July 2024. Table 1 depicts the search strategy that was devised by combining specific keywords and Medical Subject Heading (MeSH) terms with relevant Boolean operators. Additionally, a comprehensive manual search was done of the included reviews’ bibliographies.

**Table 1:** Search strategy with relevant MESH term. MESH: Medical Subject Headings

PubMed	(((("conventional"[All Fields] OR "conventionals"[All Fields]) AND ("endodontal"[All Fields] OR "endodontic"[All Fields] OR "endodontical"[All Fields] OR "endodontically"[All Fields] OR "endodontics"[MeSH Terms] OR "endodontics"[All Fields]) AND ("access"[All Fields] OR "accessed"[All Fields] OR "accesses"[All Fields] OR "accessibilities"[All Fields] OR "accessibility"[All Fields] OR "accessible"[All Fields] OR "accessing"[All Fields])) OR (("tradition"[All Fields] OR "tradition s"[All Fields] OR "traditional"[All Fields] OR "traditionals"[All Fields] OR "traditions"[All Fields]) AND ("endodontal"[All Fields] OR "endodontic"[All Fields] OR "endodontical"[All Fields] OR "endodontically"[All Fields] OR "endodontics"[MeSH Terms] OR "endodontics"[All Fields]) AND ("access"[All Fields] OR "accessed"[All Fields] OR "accesses"[All Fields] OR "accessibilities"[All Fields] OR "accessibility"[All Fields] OR "accessible"[All Fields] OR "accessing"[All Fields]))) AND (("conservancies"[All Fields] OR "conservancy"[All Fields] OR "conservancy s"[All Fields] OR "conservation"[All Fields] OR "conservational"[All Fields] OR "conservations"[All Fields] OR "conservative"[All Fields] OR "conservatively"[All Fields] OR "conservatives"[All Fields] OR "conserve"[All Fields] OR "conserved"[All Fields] OR "conserves"[All Fields] OR "conserving"[All Fields]) AND ("endodontal"[All Fields] OR "endodontic"[All Fields] OR "endodontical"[All Fields] OR "endodontically"[All Fields] OR "endodontics"[MeSH Terms] OR "endodontics"[All Fields]) AND ("access"[All Fields] OR "accessed"[All Fields] OR "accesses"[All Fields] OR "accessibilities"[All Fields] OR "accessibility"[All Fields] OR "accessible"[All Fields] OR "accessing"[All Fields]))) OR (("trusses"[MeSH Terms] OR "trusses"[All Fields] OR "truss"[All Fields]) AND ("endodontal"[All Fields] OR "endodontic"[All Fields] OR "endodontical"[All Fields] OR "endodontically"[All Fields] OR "endodontics"[MeSH Terms] OR "endodontics"[All Fields]) AND ("access"[All Fields] OR "accessed"[All Fields] OR "accesses"[All Fields] OR "accessibilities"[All Fields] OR "accessibility"[All Fields] OR "accessible"[All Fields] OR "accessing"[All Fields]))) AND (("fractur"[All Fields] OR "fractural"[All Fields] OR "fracture s"[All Fields] OR "fractures, bone"[MeSH Terms] OR ("fractures"[All Fields] AND "bone"[All Fields]) OR "bone fractures"[All Fields] OR "fracture"[All Fields] OR "fractured"[All Fields] OR "fractures"[All Fields] OR "fracturing"[All Fields]) AND ("resist"[All Fields] OR "resistance"[All Fields] OR "resistances"[All Fields] OR "resistant"[All Fields] OR "resistants"[All Fields] OR "resisted"[All Fields] OR "resistence"[All Fields] OR "resistences"[All Fields] OR "resistent"[All Fields] OR "resistibility"[All Fields] OR "resisting"[All Fields] OR "resistive"[All Fields] OR "resistively"[All Fields] OR "resistivities"[All Fields] OR "resistivity"[All Fields] OR "resists"[All Fields]))	50
Scopus	((“Truss Endodontic Access Cavity” OR “Conservative Endodontic Access Cavity” OR “Conventional Endodontic Access Cavity” OR “Traditional Endodontic Access Cavity”)) AND ((“Fracture Resistance”))	7
EBSCOHost	(((“Conventional Endodontic Access” OR “Traditional Endodontic Access”)) AND ((“Conservative Endodontic Access” OR “Truss Endodontic Access”)) AND ((“Fracture Resistance”))	14
BVS	(Conventional Endodontic Access) OR (Traditional Endodontic Access) AND (Conservative Endodontic Access) AND (Truss Endodontic Access) AND (Fracture resistance)	14
Wiley	“Traditional Endodontic Access” anywhere and “Conventional Endodontic Access” anywhere and “Conservative Endodontic Access” anywhere and “Truss Endodontic Access” anywhere and “Fracture resistance”	17
Google Scholar	(((“Conventional Endodontic Access” OR “Traditional Endodontic Access”)) AND ((“Conservative Endodontic Access” OR “Truss Endodontic Access”)) AND ((“Fracture Resistance”))	141

Selection Criteria

The inclusion criteria for this review comprised studies comparing the fracture resistance between conservative and truss endodontic access techniques and conventional or traditional endodontic access techniques. Second, the studies that involved human molars with fully developed roots were included. Lastly, only the in-vitro and ex-vivo studies were taken for the review.

The exclusion criteria for this review included studies that compared fracture resistance properties in teeth other than molars. Research involving deciduous teeth or endodontic access cavity designs other than truss and conservative access were also excluded. Additionally, studies conducted on training simulated resin teeth or animal teeth were not considered. Publications in languages other than English, as well as editorial and commentary pieces and case reports, were excluded from this study.

Data Extraction

The publication’s details, such as the year, journal name, first author’s name, databases searched, language accessed, period of the search, type of study, comparison group, quality assessment tools in the study, outcomes analyzed, the number of included studies, sample size and the type of access cavity design, were included on specific sheets that were framed.

Two independent reviewers (M.M. and A.G.) scrutinized the titles and abstracts of pertinent papers that satisfied the eligibility requirements while adhering to the tight inclusion and exclusion criteria. Any discrepancies were discussed with a third reviewer (S.S.) to be rectified. To find papers that might have been missed in the database search, the citation lists and reference lists from the included studies were additionally screened. For the studies meeting the inclusion criteria, full-text analysis was carried out.

Methodological Quality Assessment

All of the papers meeting the eligibility requirements were evaluated for study quality using a modified version of the quality assessment of in-vitro studies according to the QUIN assessment tool (risk of bias). This tool consisted of assessing the methodological quality of included studies based on six domains. Two impartial reviewers assessed the risk of bias for each domain. Any discrepancy in the quality assessment was settled by consensus.

Summary Measures and Synthesis of Results

Continuous data from eligible studies were only included in the meta-analysis. The continuous primary outcomes as well as the combined effect were assessed using the standardized mean difference (SMD) with 95% confidence interval (CI) which is equal mean difference divided by the pooled standard deviation. SMD greater than zero is considered as raised in the values whereas less than zero is regarded as a decline in the values. This effect size was considered small (0.2), medium (0.5), and large (0.8). Heterogeneity was examined by inspecting the forest plot and using the statistical test I-square.

Results

Study Selection

A total of 243 papers were obtained using the suitable mesh term from all six different search databases and protocols. Duplicate studies were excluded. The evaluation of titles and abstracts produced a total of 23 articles out of which the full text of three articles could not be retrieved.

**Figure 1:**
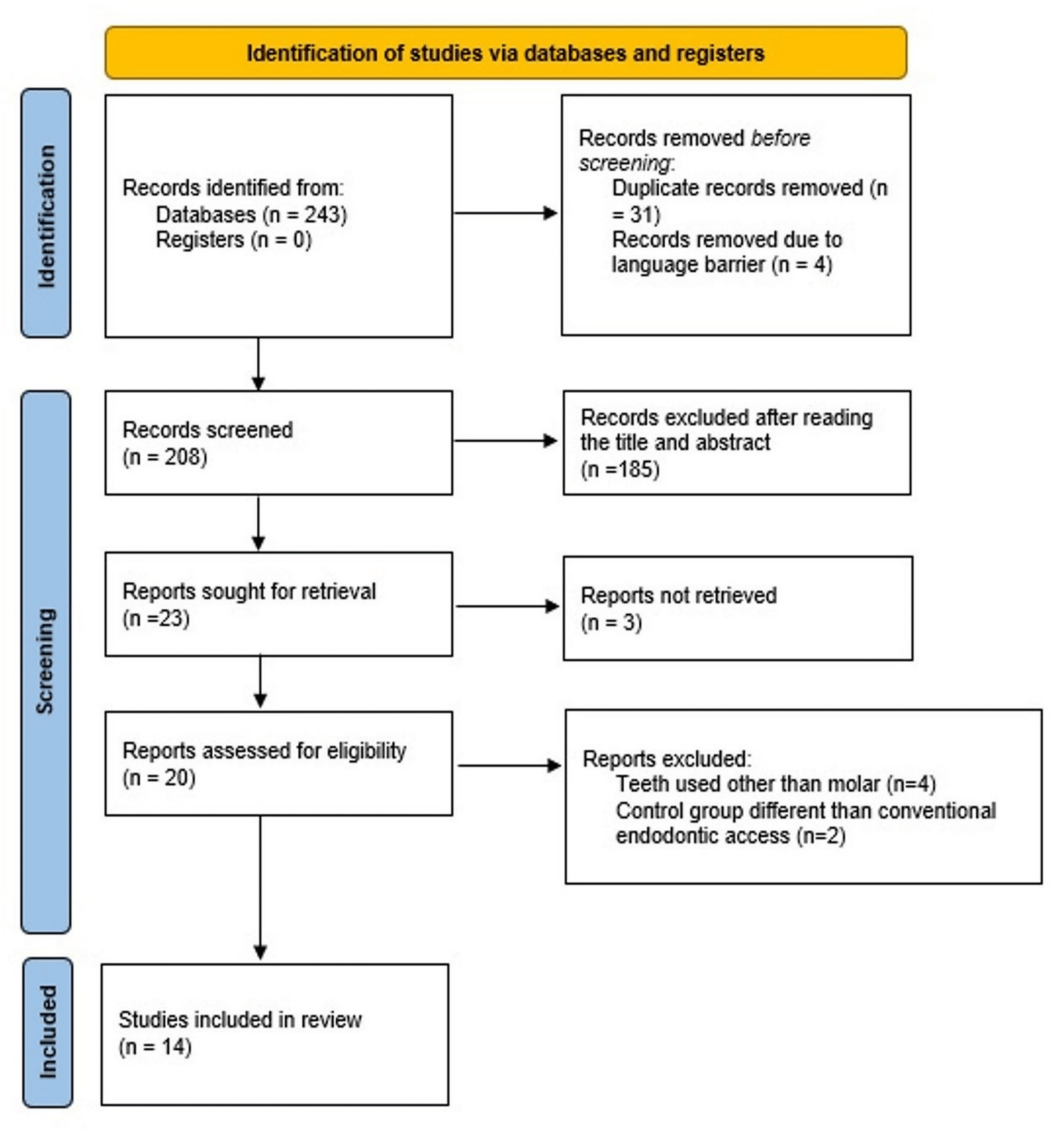
PRISMA flowchart PRISMA: Preferred Reporting Items for Systematic Reviews and Meta-Analysis The chart was prepared by Dr. Mrinalini.

Only 14 of these 23 articles met the strict inclusion requirements; therefore, this review’s analysis was limited to those studies. The comprehensive selection process and inclusion of articles are visually represented in Figure 1 through the PRISMA flow diagram.

Study Characteristics and Quality Assessment

Table 2 includes individual characteristics of 12 studies comparing the fracture resistance of conventional access with conservative access and Table 3 depicts individual characteristics of nine studies comparing the conventional access with truss access. A literature search of the relevant articles and studies was conducted from January 2000 to July 2024. All of the included studies were conducted in-vitro/ex-vivo on extracted mandibular molar teeth. The overall risk of bias calculated for the included studies’ methodological quality assessment was as follows: When all of the domains were deemed to be low risk, a score of low risk of bias was granted; when one of the domains had some issues or concerns, a score of some concerns was granted; and whenever more than two domains had issues, a score of high risk of bias was granted in the quality assessment.

**Table 2:** Characteristics of studies included in this review for comparison between conventional and conservative endodontic access.

S. No.	Author, Year	Journal	Population	Sample Size	Control	Experimental	Evaluation Criteria	Data	Outcome
1.	Patil et al., 2022 [13]	Cureus	Mandibular molars	10	Traditional endodontic access	Conservative endodontic access	Universal testing equipment	Mean ±SD	When compared to the traditional access design, conservative access demonstrated greater resistance to fracture.
2.	Santosh et al. 2021 [14]	Journal of Endodontics	Mandibular first and second molars	10	Conventional endodontic access	Conservative endodontic access	Multiaxial fatigue testing machine (Instron, Canton, MA)	Mean ±SD	Mandibular molars with conservative access and truss access demonstrated superior fracture resistance compared with traditional access.
3.	Barbosa et al, 2020 [15]	International Endodontic Journal	Mandibular molars	10	Conventional endodontic access	Conservative endodontic access	Universal testing machine (EMIC DL2000; EMIC, Sao Jos ˜ e dos Pinhais, Brazil)	Mean ±SD	There were no differences in fracture resistance across the groups.
4.	Makati et al., 2018 [16]	Journal of Conservative Dentistry	Molars	30	Conventional endodontic access	Conservative endodontic access	Universal testing machine	Mean ±SD	The conventional group had a lower mean fracture load than the conservative group (<0.001).
5.	Mustafa et al., 2020 [17]	The Open Dentistry Journal	Mandibular molars	4	Conventional endodontic access	Conservative endodontic access	Mechanical testing machine	Mean ±SD	In terms of fracture load resistance, conservative endodontic access cavity preparation showed superior results in all posterior teeth.
6.	Divyasree et al., 2022 [18]	Cureus	Mandibular molars	29	Conventional endodontic access	Conservative endodontic access	Universal testing machine	Mean ±SD	The traditional access group has significantly lower fracture resistance values than the intact, conservative access, ninja access, and truss access groups.
7.	Marinescu et al., 2020 [19]	Romanian Journal of Oral Rehabilitation	Molars	5	Conventional endodontic access	Conservative endodontic access	Multitest 5i resistance test machine	Mean ±SD	Increased fracture resistance was registered when conservative or ultraconservative access was performed, compared to the conventional one.
8.	Shadi et al., 2021 [20]	Indian Journal of Public Health Research & Development	Mandibular 1^st^ Molars	11	Conventional endodontic access	Conservative endodontic access	Universal material testing machine	Mean ±SD	NINJA, conservative, truss and traditional access group fracture resistance varied significantly with p < 0.001, but no significant difference was observed between conservative and truss endodontic access.
9.	Sabeti et al., 2018 [21]	Journal of Endodontics	Maxillary molars	16	Traditional endodontic access	Conservative endodontic access	Universal testing machine	Mean ±SD	Conservative in comparison with traditional access had no significant effect on fracture resistance.
10.	Corsentino et al., 2018 [10]	Journal of Endodontics	Mandibular first and second molar	10	Traditional endodontic access	Conservative endodontic access	Universal loading machine	Mean ±SD	Truss access does not increase the fracture strength of endodontically treated teeth in comparison with conservative and traditional endodontic access.
11.	Sudharsan et al., 2022 [22]	International Journal of Dental and Clinical Studies	Maxillary molars	20	Traditional endodontic access	Conservative endodontic access	Instron universal testing machine	Mean ±SD	Endodontically treated maxillary molars with ultraconservative “NINJA” access demonstrated enhanced fracture resistance when compared to traditional and conservative groups.
12.	Plotino et al., 2017 [9]	Journal of Endodontics	Maxillary and Mandibular molars	10	Traditional endodontic access	Conservative endodontic access	Mechanical material testing machine (LR30 K; Lloyd Instruments Ltd, Fareham, UK)	Mean ±SD	Teeth prepared using traditional endodontic access had lower fracture strength than those prepared using conservative and ninja endodontic access.

**Table 3:** Characteristics of studies included in this review for comparison between conventional and truss endodontic access.

S. No.	Author, Year	Journal	Population	Sample Size	Control	Experimental	Evaluation Criteria	Data	Outcome
1.	Patil et al., 2022 [13]	Cureus	Mandibular molars	10	Traditional endodontic access	Truss endodontic access	Universal testing equipment	Mean ±SD	Truss endodontic access showed significantly higher resistance to fracture compared to the conventional and conservative access design.
2.	Santosh et al. 2021 [14]	Journal of Endodontics	Mandibular first and second molars	10	Conventional endodontic access	Truss endodontic access	Multiaxial fatigue testing machine (Instron, Canton, MA)	Mean ±SD	Mandibular molars with conservative access and truss access exhibited superior fracture resistance compared with traditional access.
3.	Barbosa et al., 2020 [15]	International Endodontic Journal	Mandibular molars	10	Conventional endodontic access	Truss endodontic access	Universal testing machine (EMIC DL2000; EMIC, Sao Jos ˜ e dos Pinhais, Brazil)	Mean ±SD	There was no difference regarding fracture resistance among the groups.
4.	Divyasree et al., 2022 [18]	Cureus	Mandibular molars	29	Conventional endodontic access	Truss endodontic access	Universal testing machine	Mean ±SD	The traditional access group has significantly lower fracture resistance values than the intact, conservative access, ninja access, and truss access groups.
5.	Marinescu et al., 2020 [19]	Romanian Journal of Oral Rehabilitation	Molars	5	Conventional endodontic access	Truss endodontic access	Multitest 5i resistance test machine	Mean ±SD	Increased fracture resistance was registered when conservative or ultraconservative access was performed, compared to the conventional one.
6.	Shadi, 2021 [20]	Indian Journal of Public Health Research & Development	Mandibular 1^st^ Molars	11	Conventional endodontic access	Truss endodontic access	Universal material testing machine	Mean ±SD	There was a significant difference between ninja, conservative, truss, and traditional access groups with (p < 0.001) and no significant difference between conservative and truss groups.
7.	Corsentino et al., 2018 [10]	Journal of Endodontics	Mandibular first and second molar	10	Traditional endodontic access	Truss endodontic access	Universal loading machine	Mean ±SD	Truss access does not increase the fracture strength of endodontically treated teeth in comparison with conservative and traditional endodontic access.
8.	Abou-Elnaga et al., 2019 [23]	Journal of Endodontics	Mandibular first molars	12	Traditional endodontic access	Truss endodontic access	Computer-controlled material testing machine (Model 3345; Instron Industrial Products, Norwood, MA)	Mean ±SD	The truss access cavity preparation improved the fracture resistance of endodontically treated teeth.
9.	Saberi et al., 2020 [24]	Clinical, Cosmetic, and Investigational Dentistry	Mandibular first and second molars	10	Traditional endodontic access	Truss endodontic access	Universal testing machine	Mean ±SD	Truss endodontic access enhances the fracture strength of endodontically treated teeth under thermal stresses.

Eleven out of fourteen studies that were included in this evaluation had a low risk of bias and three showed moderate risk of bias according to the parameters evaluated during the quality assessment, which are displayed in Table 4.

**Table 4:** Risk of bias for included studies using the QUIN assessment tool. QUIN: Quality Assessment Tool For In Vitro Studies

Author, Year	Detailed Explanation for Sample Size Calculation	Tooth Standardization	Control Group (Conventional Access)	Whether the Tooth Was Restored or Not	Outcome Evaluation	Statistical Analysis	Risk of Bias
Patil et al., 2022 [13]	X	V	V	V	V	V	Low
Santosh et al. 2021 [14]	X	V	V	V	V	V	Low
Barbosa et al, 2020 [15]	V	V	V	X	V	V	Low
Makati et al., 2018 [16]	X	V	V	V	V	V	Low
Mustafa et al., 2020 [17]	X	V	V	X	V	V	Moderate
Divyasree et al., 2022 [18]	X	V	V	V	V	V	Low
Marinescu et al., 2020 [19]	X	V	V	V	V	V	Low
Shadi et al., 2021 [20]	X	V	V	X	V	V	Moderate
Sabeti et al., 2018 [21]	X	V	V	X	V	V	Moderate
Corsentino et al., 2018 [10]	X	V	V	V	V	V	Low
Sudharsan et al., 2022 [22]	X	V	V	V	V	V	Low
Plotino et al., 2017 [9]	X	V	V	V	V	V	Low
Abou-Elnaga et al., 2019 [23]	X	V	V	V	V	V	Low
Saberi et al., 2020 [24]	X	V	V	V	V	V	Low

Outcome of Meta-analysis

By assessing the similarities among the included research, a meta-analysis was conducted. Meta-analysis was done using the statistical software STATA version 14. Quantitative data synthesis was carried out to combine equivalent results. We applied the random effect method to pool the SMD across the studies because heterogeneity among the studies was high. The studies were used to extrapolate the number of specimens in each group, the mean, and the standard deviation related to the fracture resistance of traditional, conservative, and truss endodontic access. The subgroup analysis according to type of tooth, type of material, and risk-of-bias quality assessment of studies was planned but able to be done only by types of teeth because of an inadequate number of studies (at least two studies per group). Sensitivity analysis leave-one-out was used to assess the influence of the individual survey on the overall pooled effect. The publication bias was evaluated using the funnel plot and tested using the Egger and Begg statistical test.

Twelve studies comparing fracture resistance of conventional with conservative access and nine studies comparing conventional with truss access that provided continuous data were included in this meta-analysis. The mean difference is used in this analysis as a measure of effect size. The computations involved generating an index by dividing the mean difference between each research by the standard deviation of that study (SMD). The SMD, which was determined independently for each trial, was comparable across all of the investigations. Using the I2 value, which indicates low, medium, and high statistical heterogeneity at 25%, 50%, and 75%, respectively, statistical heterogeneity between studies was examined.

**Figure 2:**
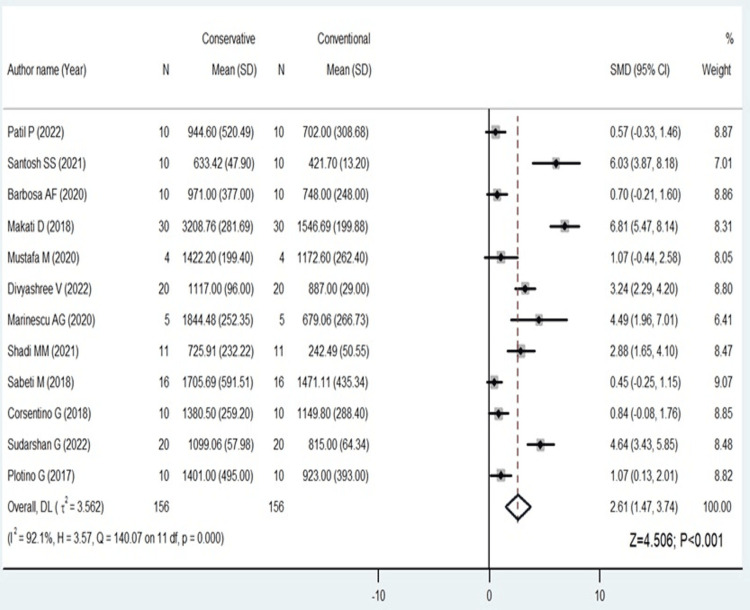
Forest plot comparing conventional and conservative endodontic access [9,10,13-22] The image was drawn by Dr. Mrinalini.

The analysis found that when the fracture resistance was examined for conventional and conservative endodontic access cavities, the forest plot displayed statistically significant results with SMD 2.61 (95% 1.47 to 3.74); p<0.001) favoring conservative endodontic access cavities (Figure 2).

**Figure 3:**
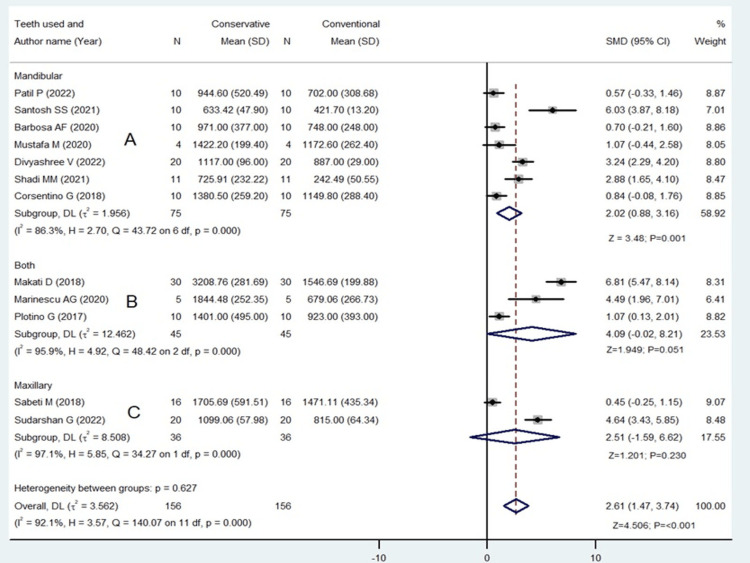
Subgroup analysis for conservative versus conventional access [9,10,13-22] The image was drawn by Dr. Mrinalini.

The subgroup according to teeth revealed that only mandibular was found to be significant with SMD 2.02 (95% CI: 0.88 to 3.16; p=0.001 (Figure 3).

**Figure 4:**
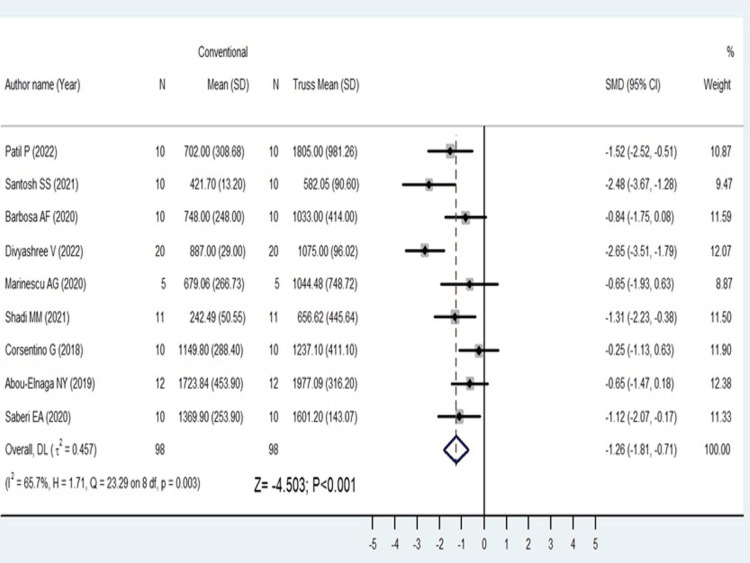
Forest plot comparing conventional and truss endodontic access [10,13-15,18-20,23,24] The image was drawn by Dr. Mrinalini.

The comparison of fracture resistance between conventional and truss access also gave significant results favoring truss access with SMD -1.26(95%CI: -1.81 to 0-0.71; p<0.001) (Figure 4).

There was a high amount of statistical heterogeneity among the included studies for conservative and conventional and marginal in the conventional and truss endodontic access (I^2^ values = 92% and 65.7%, respectively).

**Figure 5:**
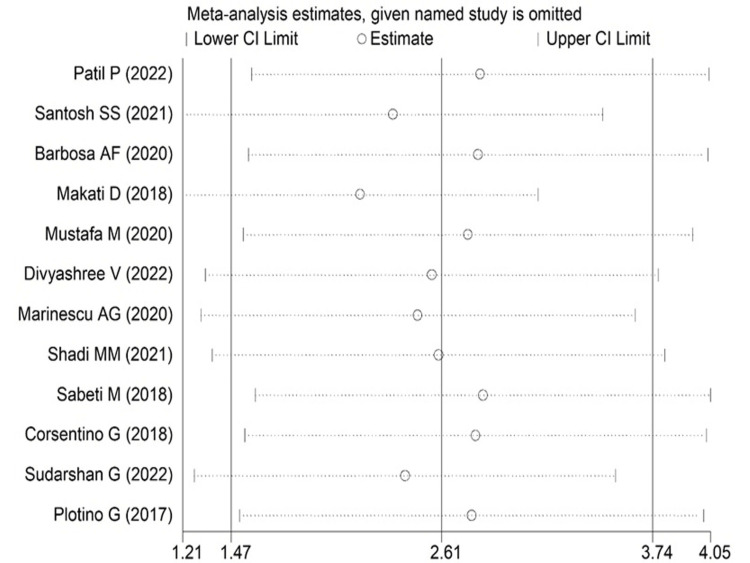
Leave-one-out graph showing the change in the pooled estimate after leaving out exactly one study for the conventional versus conservative group [9,10,13-22] The image was drawn by Dr. Mrinalini.

**Figure 6:**
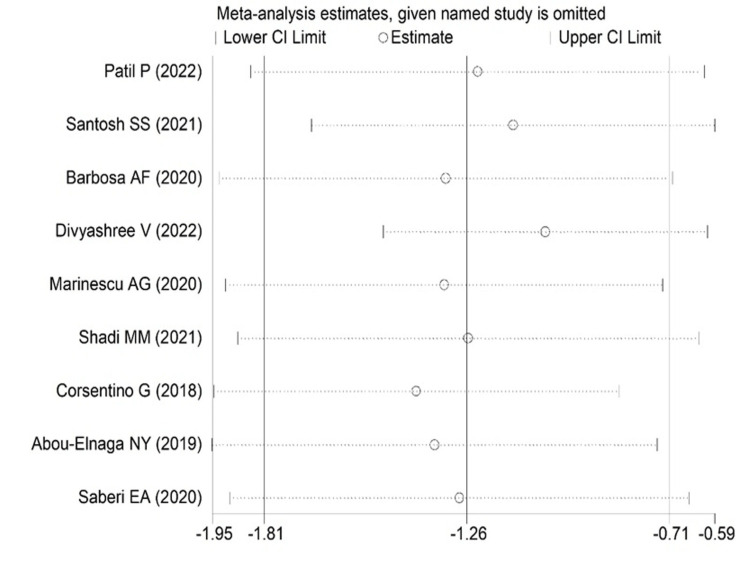
Leave-one-out graph showing the change in the pooled estimate after leaving out exactly one study for the conventional versus truss group [10,13-15,18-20,23,24] The image was drawn by Dr. Mrinalini.

In addition, the random effect of the sensitivity analysis, leave-one-out method revealed the study results were stable, except one or two studies in the conservation and conventional methods comparison and also in the conventional and truss methods were marginally influenced by the pooled effect size. However, pooled SMD was still found to be statistically significant in both comparisons (Figures 5-6). Thus, results seem to be quite robust even after the high heterogeneity.

The funnel plot showed no publication bias for the mandibular teeth in the conservative and conventional as well as conventional and truss endodontic, this was also verified by the Egger statistical test p=0.144 for conservative and conventional p=0.689 for conventional and truss endodontic.

Discussion

The primary goal of access cavities is to preserve the healthy dental structure as much as feasible. One pertinent explanation for fractures in teeth with root canal fillings is the loss of tooth structure during access cavity preparation [25].

Fundamentally in endodontics, maintaining strength and stiffness that resists structural deformation becomes the goal of every single restorative therapy. Traditional endodontic access involves the complete removal of the pulp roof chamber.

The idea of “extension for prevention” in traditional endodontic access cavities is used to facilitate the treatment and prevent dentin from getting in the way of obtaining straight-line access. Unfortunately, this leads to significant tooth structure loss [26]. It can be stated that the conservative endodontic access cavity idea aims to maintain the occlusal tooth structure by minimizing the access cavity. This concept gained wide popularity leading to the design of newer ultraconservative designs which in turn allows the retention of more dental structures including cervical dentin and roof chamber. The preservation of PCD, soffit, and the 3-D ferrule is attributed to the long-term survival of the tooth [27, 28, 29].

Conservative access cavity preparation is characterized by smoothly convergent axial walls to the occlusal surface only to an extent that facilitates the identification of canal orifices, preserving part of the pulp roof chamber [5]. Whereas truss endodontic access conserves the dentinal bridge present between two or more small cavities prepared to locate the canal orifice in each root of multi-rooted teeth [30]. The present systematic review of in-vitro studies assessed and compared the effect of conventional, conservative, and truss endodontic access on fracture resistance of permanent molar teeth.

From 243 studies obtained after an electronic search through six different databases, 12 articles comparing fracture resistance of conservative and conventional access, and nine articles comparing truss and conventional access were taken into consideration based on inclusion and exclusion criteria. The final studies further underwent risk of bias and were categorized into low/moderate/high-risk categories.

Out of the total of 14 studies, Corsentino et al., 2018 and Barbosa et al., 2020 concluded no significant difference in fracture resistance [10,15].

All the other studies showed conservative and truss access to be better than conventional access in terms of fracture resistance.

Silva et al., in the year 2017 evaluated the fracture resistance of conservative access with conventional access for all the groups of teeth (incisors, premolars, and molars) in a systematic review. Out of 180 articles, six satisfied their inclusion requirements. While the other three had a moderate risk of bias, three of them had a low risk. Silva et al. discovered that, although half of the included publications showed an improvement in fracture resistance when using conservative access in comparison to performing conventional access, the other half of the included articles revealed no significant difference between the two groups [31].

Another systematic review conducted by Saeed et al., in the year 2021 revealed that there is insufficient data to definitively state if conservative access offers greater fracture resistance than conventional access [7].

Motiwala et al. conducted a systematic review in the year 2022 where 14 articles were taken for systematic review and 10 articles for meta-analysis. The data collection was done till September 2020. The article concluded that the conservative endodontic access cavity had the highest fracture resistance compared to the truss and conventional endodontic access. [32]

The conservative and truss access cavity presents various challenges to clinicians. The accessibility of the offending tooth is the fundamental issue with molar teeth. In a situation where accessibility is restricted, these minimal access cavity designs might be challenging. Detecting all canals, extracting pulp tissues from pulp horns, removing debris and necrotic material, and resolving any procedural complications that may emerge due to a limited access cavity are other difficulties that clinicians often encounter [31, 33, 34]. Clinicians should consider adopting conservative or truss designs to enhance tooth fracture resistance, especially in cases where preserving tooth structure is crucial.

However, one significant drawback of the studies examined in this review is that they were all conducted either in-vitro or ex-vivo. As a result, caution must be taken when implementing the findings in therapeutic settings. The considerable degree of heterogeneity found among the included studies was another limitation of this review. The high level of heterogeneity in both the groups with I2 values of 95% and 85% for comparison between conventional and conservative as well as conventional and truss endodontic access respectively could be attributed to the type of molar selected, time of analysis of whether fracture resistance was done in freshly extracted teeth or for the teeth stored in a medium and difference in restorative material used.

## Conclusions

In recent years, there has been a notable surge in the adoption of conservative minimal access cavity designs. According to the results of the current analysis, conservative and truss endodontic access cavity designs provide superior fracture resistance than traditional endodontic access within the confines of the study. This is attributed to the conservation of tooth structure including PCD, soffit, and 3D ferrule in the newer access cavity designs. However, further analysis is required to assess the risk-benefit ratio of contracted access cavity designs and assess the long-term effects of minimal preparations on treatment outcomes in an in-vivo environment. Also, future studies should include in-vivo trials to validate these findings and explore the long-term effects of different access cavity designs on tooth survival and restoration success.
